# Neem biomass derived carbon quantum dots synthesized via one step ultrasonification method for ecofriendly methylene blue dye removal

**DOI:** 10.1038/s41598-024-59483-9

**Published:** 2024-04-27

**Authors:** Zakriya Waseem Basha, Sarangapani Muniraj, Annamalai Senthil Kumar

**Affiliations:** 1 P.G. & Research Department of Chemistry, RKM Vivekananda College (Autonomous), Mylapore, Chennai, 600004 India; 2 Nano and Bioelectrochemistry Research Laboratory, Carbon Dioxide and Green Technologies Research Centre and Department of Chemistry, School of Advance Science, Vellore Institute of Technology University, Vellore, Tamil Nadu 632 014 India

**Keywords:** Biomass derived carbon dots (BCDs), One step ultrasonification, Methylene blue dye removal, In-situ precipitation, Surface regeneration, Environmental sciences, Chemistry

## Abstract

This article presents a one-step ultrasonication technique for generating biomass carbon dots (BCDs) from neem bark (Azadirachta indica) powder. The BCDs were characterized using modern techniques such as UV–Vis, FTIR, Raman, XRD, HRTEM, FESEM, EDAX, and Zeta potential analyses. Unlike traditional nanocomposite bed systems, this study utilized BCDs as a liquid-phase adsorbent for the regenerative adsorption of the environmentally harmful dye, methylene blue (MB), through an in-situ precipitation reaction. This involved the formation of BCDs-MB adduct via an electrostatic mechanism. The adsorption capacity and percentage of removal were remarkable at 605 mg g^–1^ and 64.7% respectively, exceeding various solid-based adsorption methods in the literature. The Langmuir isotherm and pseudo-second-order kinetics model provided an excellent fit for this system. The calculated thermodynamic parameter, Gibbs free energy change (ΔG) was negative, indicating a spontaneous, exothermic, and physisorption-based mechanism. The regenerative capacity of our system was further demonstrated by successfully extracting and recovering the MB dye (64%) using ethyl alcohol as the solvent. This method provides an efficient means of recovering valuable cationic organic dye compounds from contaminated environments.

## Introduction

Industrial waste-associated organic dyes, such as MB, rhodamine B, crystal violet, and others, are increasingly contributing to water pollution^1,2^. These organic dyes pose a significant risk to human and animal health, causing harm to organs like the kidneys, liver, brain, and reproductive and central nervous systems, as well as leading to mutations and carcinogenic effects^3,4^. Furthermore, they have substantial adverse effects on the environment, including the inhibition of the photosynthetic process, reduction of sunlight penetration^5^, consumption of dissolved oxygen, and diminished recreational value of water bodies. Currently, various methods are employed to detoxify wastewater containing these organic dyes, including coagulation/flocculation^6,7^, membrane filtration^8^, advanced oxidation^9^, ozonation^10^, photocatalytic degradation^11^, biodegradation^12^, and adsorption^13^. Among these methods, adsorption-based processes are deemed the most suitable due to their high removal efficiency, use of cost-effective adsorbents, and ease of operation^14–18^. In contrast, other techniques, such as photocatalytic degradation, result in the emission of secondary pollutants like CO_2_, phenol, hydrocarbons, and other organic compounds^19,20^.

Regarding adsorption-based dye removal, sorbents like microporous silica^21^, silica nanoparticles^22^, carbon composites^23^, and biochar^24^ offer numerous advantages, including high adsorption capacity, potential for regeneration and reuse, but also come with several drawbacks, such as the use of hazardous precursor chemicals, high energy requirements, high costs, the need for expensive equipment, challenging operating conditions, and sample toxicity. To address these challenges, carbon dots were synthesized utilizing biomass as a precursor. This approach offers numerous advantages, including generation of a high carbon content coupled with polar functional groups that enhance adsorption capacity. Moreover, biomass serves as an easily accessible, environmentally friendly, and cost-effective alternative, avoiding the use of harmful chemicals in the synthesis process. Overall, biomass stands out as an excellent precursor for carbon dot production, offering a renewable resource with multifaceted benefits. Carbon dots and carbon-based nanoparticles are utilized to construct, modify, and enhance the electrical conductivity of nanocomposite beds^25,26^. However, there have been no reports of carbon dots being used as the sole adsorbent material precursor. In this study, we introduce a novel and straightforward method for BCDs suitable for the efficient and regenerative removal of organic dyes, with MB serving as the model system, from aqueous solutions.

Carbon dots, composed of angstrom-sized polyaromatic carbon cores layered with a graphitic lattice, exhibiting sp^2^/sp^3^ hybridization, and possessing zero-dimensional structures with sizes less than 20 nm, are cost-effective and efficient fluorescent materials. They have found diverse applications in science and technology, including cellular imaging, chemical sensing, biochemical sensing, and electrical conductivity^25–33^. Various synthesis methods, such as chemical oxidation^34^, hydrothermal processes^35–37^, microwave-assisted techniques^38^, and electrochemical approaches^39^, have been employed using organic compounds as precursors. However, these methods often suffer from drawbacks such as high energy consumption, complex processing steps, uncontrollable reactions, the use of corrosive chemicals, and limited commercial feasibility^40^. In our study, we introduce a one-step ultrasonication method for synthesizing BCDs, which is a simple, cost-effective, and scalable approach suitable for large-scale industrial production.

Recently, the environmental impact of organic dyes has become a pressing concern. The annual production of organic dyes exceeds 700,000 tonnes, with around 20% of these dyes ending up in industrial wastewater^5^. To address this issue, various methods for dye degradation, including chemical^41^, photochemical^42^, and electrochemical processes^43^, have been reported. Researchers have extensively employed photocatalysis for the removal of organic dyes owing to its myriad advantages. These benefits encompass consistent chemical stability, affordability, simplicity in sample preparation, and prompt outcomes. Importantly, it should be noted that the degradation of dyes frequently leads to the emission of hazardous compounds, including carbon dioxide, phenol, hydrocarbons, and other organic molecules. These substances are categorized as greenhouse gases and carcinogens, thus posing significant risks to both human health and the environment^19,20,44,45^. Alternatively, a more environmentally friendly approach is selective dye regeneration rather than decomposition. It's worth noting that there is a lack of literature focusing on the regeneration of organic dyes. In our work, we present an innovative approach using BCDs for environmental pollution remediation and regeneration of toxic organic dyes, with the model system being MB dye.

**Figure 1:**
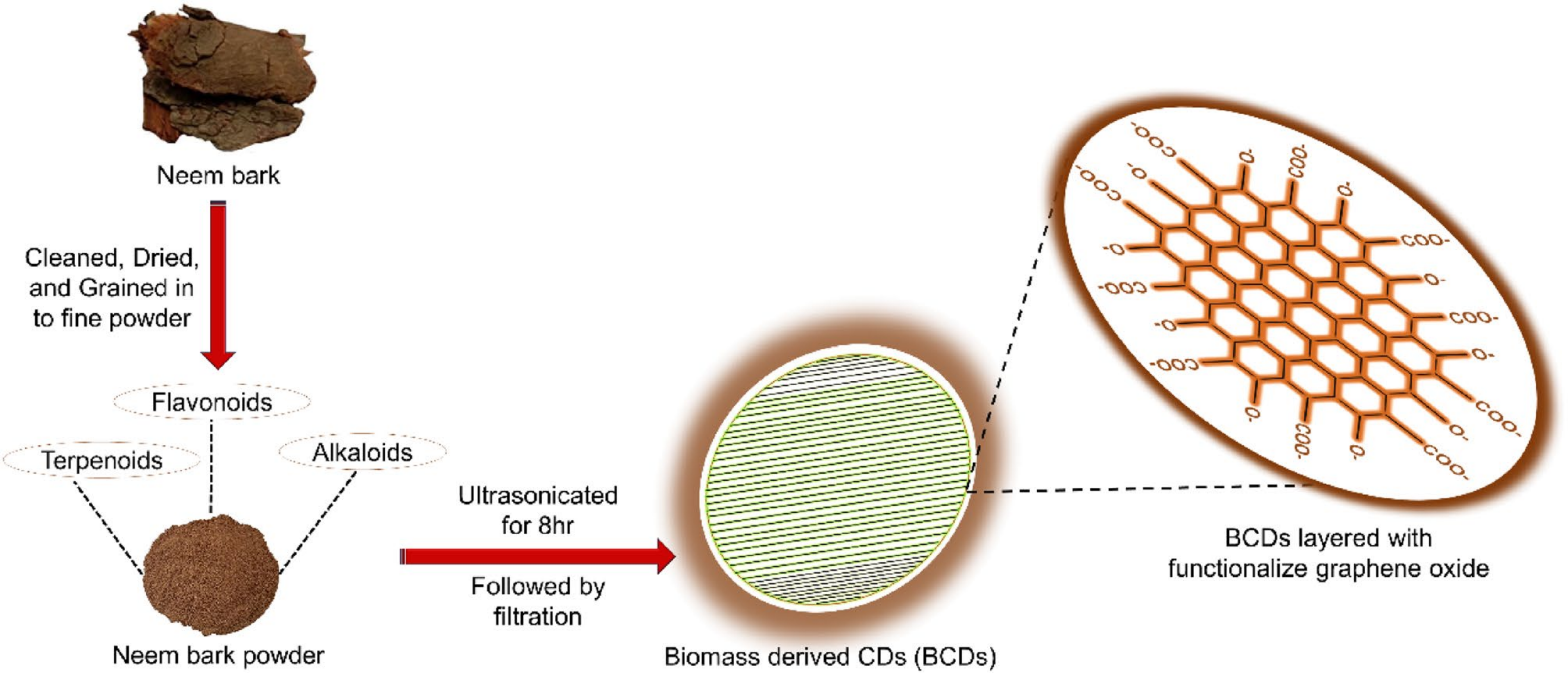
Synthesis of BCDs using ultrasonication method.

In this study, we utilized neem bark powder as a precursor to synthesize BCDs through a one-step ultrasonication procedure, as illustrated in Fig. 1. Neem bark contains a variety of phytochemicals, including alkaloids, flavonoids, terpenoids, anthraquinones, cardiac glycosides, phytosterols, polyphenols, and saponins, which serve as source materials for BCDs formation^46^. We conducted adsorption studies to determine the maximum adsorption capacity and dye removal percentage. The experimental results revealed a maximum adsorption capacity of 605 mg g^–1^ and a dye removal percentage of 64.70%. These values surpass those reported in most of the literature for conventional materials (Table 1)^21,47–54^.

**Table 1:** Comparative evolution of adsorption capacity with other literature compound.

Adsorbent	Adsorption capacity (mg g^–1^)	Reference
1. Magnetic ball milled AC	304.2	^46^
2. Microporous silica (MS)	308	^47^
3. Organo-microporous silica (Organo- MS)	308	^47^
4. Natural clay	322.58	^21^
5. Manganese-modified lignin biochar (BC-MnO_2_)	355.96	^48^
6. Nitrogen and sulfur doped carbon quantum dots/hexagonal mesoporous silica (N,S-CQD/HMS)	370.4	^49^
7. silica nanoparticles grafted with copolymer of acrylic acrylamide (SAA)	375.9	^50^
8. Thiourea-modified poly(acrylonitrile-co-acrylic acid) (TA-p(AN-co-AA))	440.8	^51^
9. Magnetic ball milled BC	500.2	^46^
10. Magnetic hydroxyapatite nanorods	531.0	^52^
11. Nano–silica (nSiO_2_)	547.2	^53^
12. Microwave modified nano–silica (MW-nSiO_2_)	679.9	^53^
13. BCDs	605	This work

Furthermore, we employed the Langmuir isotherm and the pseudo-second-order kinetics models to elucidate the kinetics and mechanisms underlying MB dye removal. We propose that electrostatic attraction between water-soluble anionic BCDs and cationic MB dye leads to the formation of a water-insoluble adduct. In summary, our work presents a simple, environmentally friendly approach using ultra-low-cost BCDs for environmental pollution remediation through regenerative dye removal.

## Experimental methods

Methylene blue dye (C_16_H_18_ClN_3_S, Mw = 319.85 g mol^–1^, CAS: 61-73-4, Merck-India) sodium hydroxide (NaOH, Mw = 40 g mol^–1^, CAS: 1310-73-2, Merck-India), hydrochloric acid (HCl, Mw = 36.46 g mol^–1^, 35%, CAS: 7647-01-0, Merck-India), sodium chloride (NaCl, Mw = 58.44 g mol^–1^, CAS: 7647–14-5, Merck-India), potassium chloride (KCl, Mw = 74.55 g mol^–1^, CAS: 7447-40-7, Merck-India), lead nitrate (PbNO_3_, Mw = 331.21 g mol^–1^, CAS: 10099-74-8, Merck-India), nickel chloride (NiCl_2_, Mw = 129.60 g mol^–1^, CAS: 7718-54-9, Merck-India), copper sulphate (CuSO_4_, Mw = 159.61 g mol^–1^, CAS: 7758-98-7, Merck-India), ferrous sulphate (FeSO_4_, Mw = 278.01 g mol^–1^, CAS: 7782-63-0, Merck-India), mercuric sulphate (HgSO_4_, Mw = 296.65 g mol^–1^, CAS: 7783-35-9, Merck-India), sodium acetate (CH_3_COONa, Mw = 83.03 g mol^–1^, CAS: 127-09-3, Merck-India), cadmium carbonate (CdCO_3_, Mw = 172.42 g mol^–1^, CAS: 513-78-0, Merck-India) and barium chloride (BaCl_2_, Mw = 208.23 g mol^–1^, CAS: 10361-37-2, Merck-India) are utilized. A deionized double distilled water was used throughout the study whereas the deionized double distilled water purchased from the Andavar distilled water co, Saidapet, Chennai. The temperature of the adsorption process maintained at room temperature i.e. 28 ± 2 °C.

### Synthesis of biomass-derived carbon dots

The wild raw neem bark was sourced from Ramakrishna Mission Vivekananda College, Chennai, India. The fielded studies of plants experiment complied with relevant institutional, national, and international guidelines and legislation. About 200 g of the neem bark were cut into nearly uniform pieces, cleaned with hot- double-distilled water to get rid of foreign material like soil, pathogens and dried naturally in the sun for 10 to 15 days before being powdered using the graining technique. About 4 g of neem bark powder was dispersed in 100 ml of double distilled water and ultrasonically treated (40 kHz) for 8 h. A dark brown colloidal solution obtained was subjected to centrifuging for 30 min at 10,000 rpm to remove unreactive and heavy larger size particles, followed by filtering using a 0.22 µm cellulose membrane filter. The resultant supernatant clear brown aqueous solution (BCDs) was collected and utilized for further characterization and regenerative dye removal applications (Fig. 1). The yield of the BCDs was found to be 36.25% and the calculation are as given below:

$$\mathrm{\% \,\,of \,\,Yield }\left(\mathrm{BCDs \,\,formed}\right)= \frac{\mathrm{Amount\,\, of \,\,BCDs \,\,formed \,\,after \,\,the \,\,process}}{\mathrm{Amount \,\,of \,\,neem\,\, bark\,\, initially\,\, taken}} \times 100.$$

$$\mathrm{\%\,\, of \,\,Yield }\left(\mathrm{BCDs \,\,formed}\right)= \frac{1.45}{4} \times 100=36.25\mathrm{ \%}.$$

### Preparation of sample for SEM-EDAX analysis

A drop of BCDs was taken in a capillary tube and diluted in 10 ml of water which was uniformly distributed in aluminium foil and kept for drying overnight at room temperature. The dried BCDs coated aluminium foil was utilised for SEM- EDAX analysis (Fig. S1).

## Results and discussion

### Physiochemical characterization of BCDs

**Figure 2:**
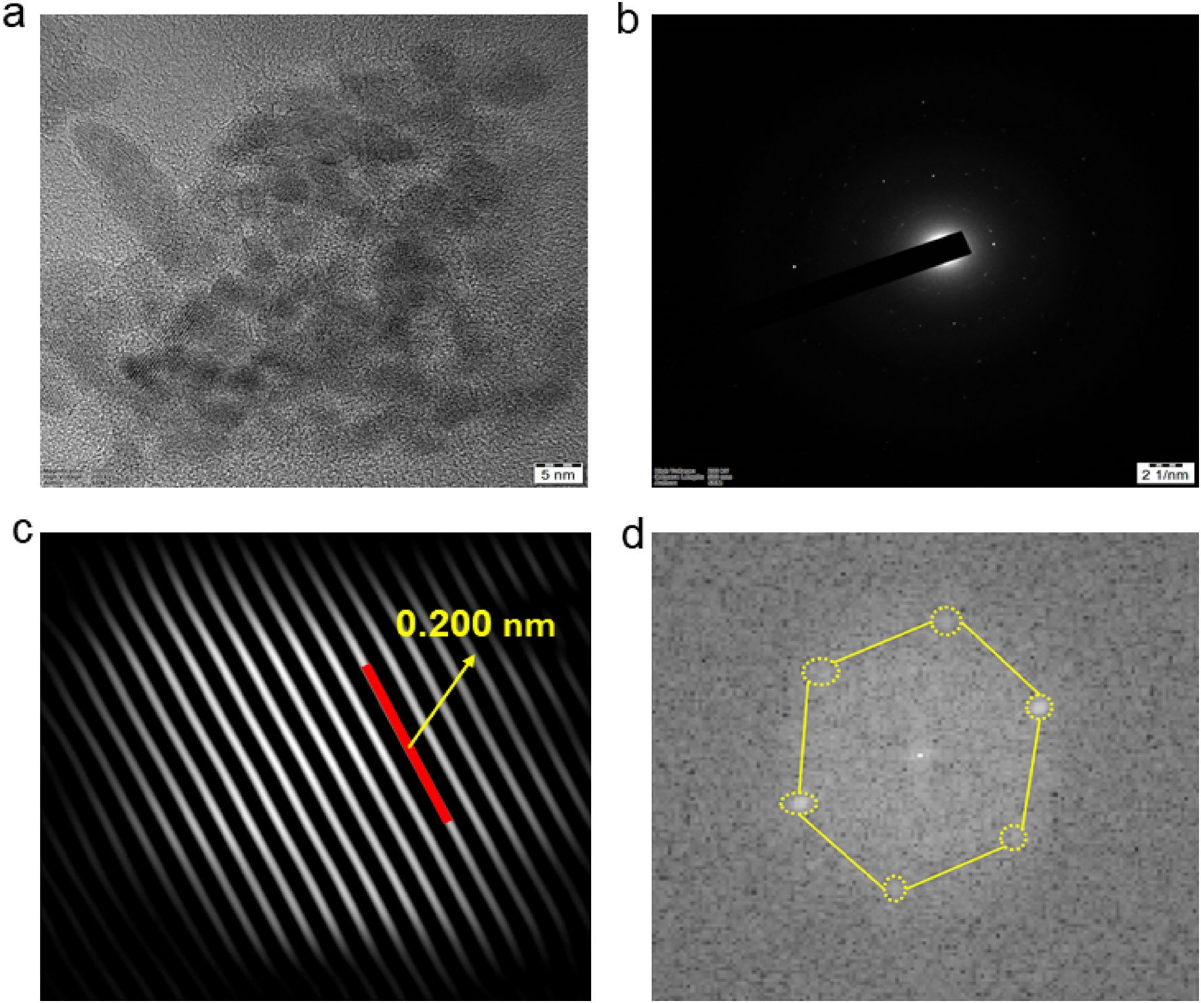
(**a**) The TEM image depicts the homogeneous distribution of BCDs having an average size of 5 nm, (**b**) The selected area electron diffraction (SAED) Patten of BCDs observed at 21 nm, (**c**) The fringes of BCDs spacing between them is 0.200 nm, (**d**) The fast fourier transform (FFT) image of BCDs.

Figure 2a presents a transmission electron microscopy (TEM) analysis of BCDs, revealing quasi-spherical particles with a non-dispersive nature, ranging in size from 2 to 10 nm, without any signs of aggregation. The selected area electron diffraction (SAED) pattern, detected at 21 nm, indicates a poorly crystalline nature of the material, likely due to the amorphous structure of graphene oxide (Fig. 2b). A high-resolution TEM image of the particles exhibits clearly defined fringes with a spacing of 0.200 nm, attributed to the graphitic structure on the particle surface and corresponding to the (100) plane of graphite on the surface (Fig. 2c). The Fast-Fourier Transform (FFT) picture reveals the hexagonal arrangement of carbon atoms in the graphitic network (Fig. 2d)^55–59^.

**Figure 3:**
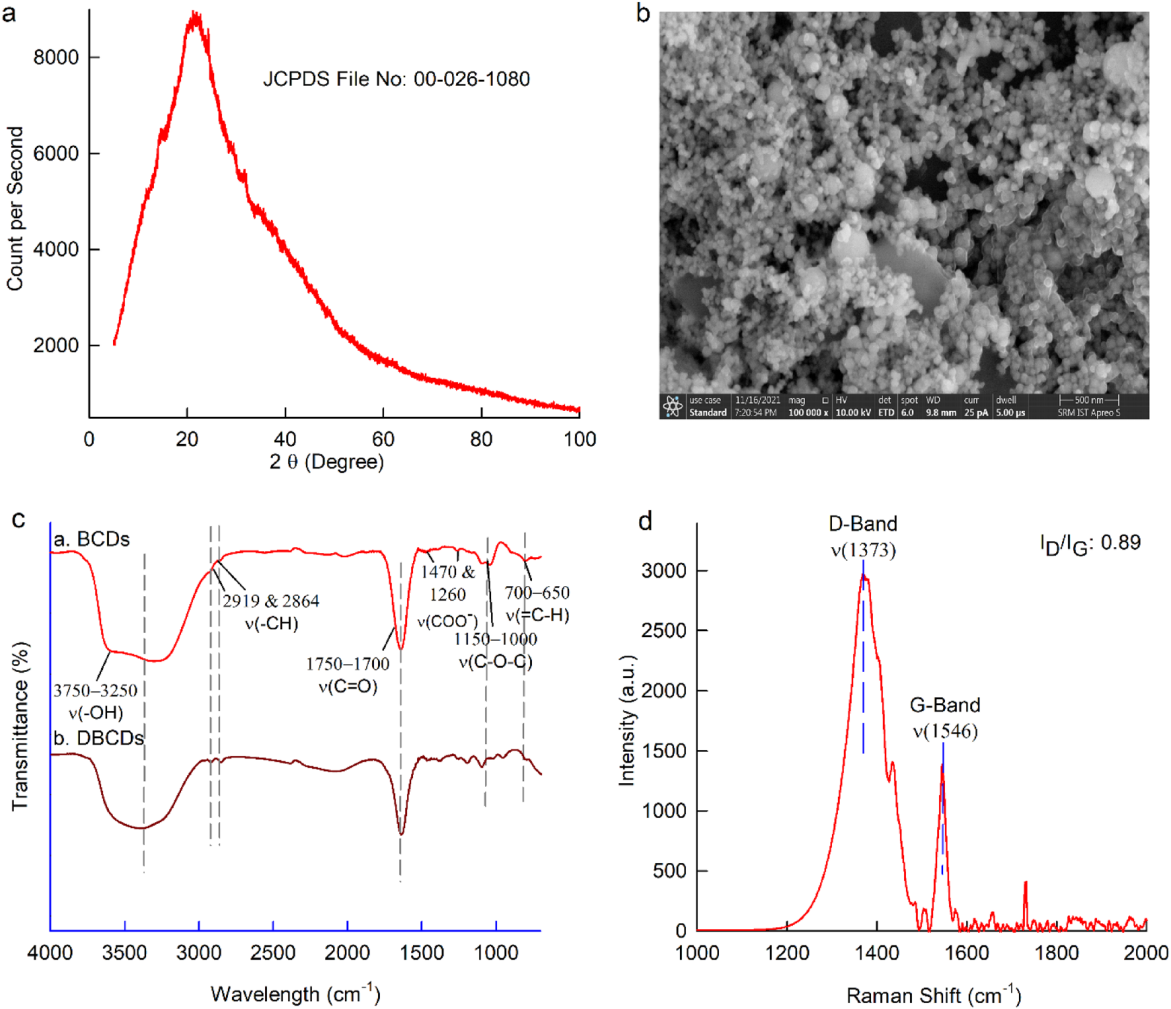
(**a**) The XRD pattern of BCDs shows a wide diffraction peak around 2θ = 21.18°, (**b**) The FESEM image of BCDs illustrates the uniformly distributed spherical shape sized above 10 nm, (**c**) The FTIR spectrum of a. BCDs and b. dried BCDs (DBCDs), (**d**) The Raman spectrum of BCDs having bands at 1373 cm^–1^ (D-Band) and 1546 cm^–1^ (G-Band).

In Fig. 3a, we observe a typical X-ray diffraction (XRD) pattern of lyophilized BCDs, which displays a broad peak at 2θ = 21.18° corresponding to the (002) lattice spacing were formed of polyaromatic C domains surrounded with amorphous carbon network^60^. This observation closely matches the XRD pattern of amorphous carbon materials with JCPDS File No. 00-026-1080, supporting the amorphous nature of the BCDs. Further examination through Field Emission Scanning Electron Microscopy (FESEM) in Fig. 3b shows spherical, monodispersed particles with a size of approximately 10 nm, consistent with the size observed in the TEM analysis. In Fig. S1, Energy-Dispersive X-ray Analysis (EDAX) of the BCDs sample reveals a composition of 64.16% carbon and 35.85% oxygen.

To confirm the presence of various carbon–oxygen functional groups, we conducted FTIR spectroscopy, as depicted in Fig. 3c. The specific IR signals corresponding to functional groups, such as –COO^–^ (1470 cm^–1^ and 1260 cm^–1^), C=O (1750–1700 cm^–1^), C–O–C (1150 cm^–1^ and 1000 cm^–1^), –CH (2919 and 2864 cm^–1^),  =CH (700–650 cm^–1^), and –OH (3750–3250 cm^–1^), are evident. These results indicate the presence of several carbon–oxygen functional groups in the BCDs, attributed to the graphene oxide core network structure. To eliminate any influence of chemisorbed water on the chemical structure of BCDs, we subjected the BCDs to air drying under vacuum at approximately 50 °C for 5 days (referred to as DBCDs = Dried BCDs). As shown in Fig. 3c, the FTIR response of DBCDs exhibits qualitatively similar signals to those of the BCDs, indicating that chemisorbed water has no significant impact on the chemical structure of the BCDs^61^.

To substantiate the presence of the graphitic structure detected in the TEM images and FTIR results, we performed Raman spectroscopy of BCDs, considered a fingerprint analysis for authenticating the graphene oxide core structure^62^. In Fig. 3d, the Raman spectrum of BCDs reveals two characteristic bands at 1373 cm^–1^ (D-band), associated with sp^3^ hybridized carbon defects (graphitic disorder) due to A_1g_ vibrating phonons, and 1546 cm^–1^ (G-band), corresponding to sp^2^-bonded carbon atoms in a two-dimensional hexagonal lattice due to the E_2g_ vibration mode of the graphitic structure. The qualitative nature of BCDs is evaluated using the intensity ratio of the D-band to the G- band (I_D/_I_G_), resulting in a calculated value of 0.89, indicative of a distorted graphite nature (amorphous nature). The cumulative Raman results of BCDs reveal sp^3^ hybridized carbon defects alongside sp^2^ carbon–oxygen functionalized graphite carbon^55,56,63,64^.

**Figure 4:**
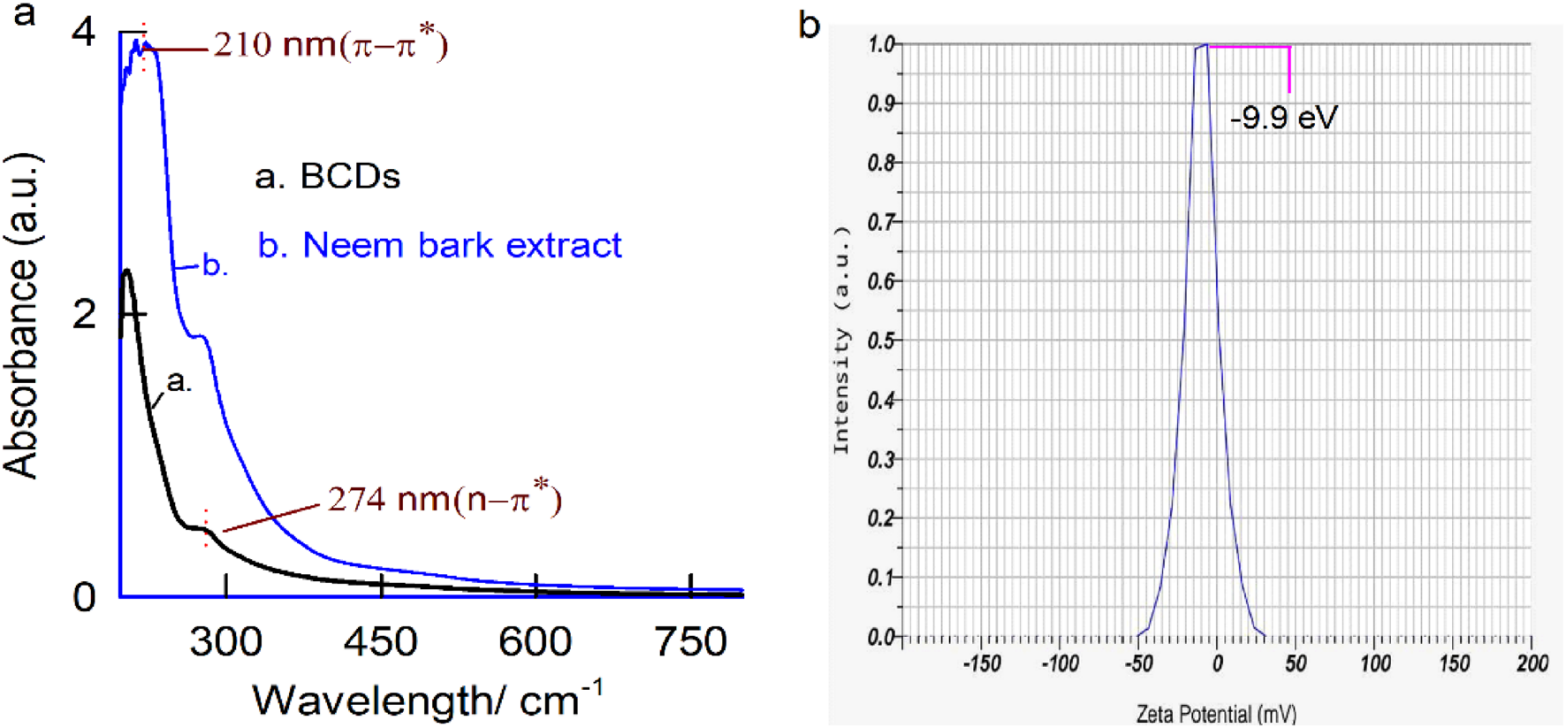
(**a**) The UV spectrum of BCDs having absorption peaks at λ_max_ = 210 & 274 nm, (**b**) The zeta potential measurement of BCDs showing a surface charge peak at − 9.9 eV.

**Figure 5:**
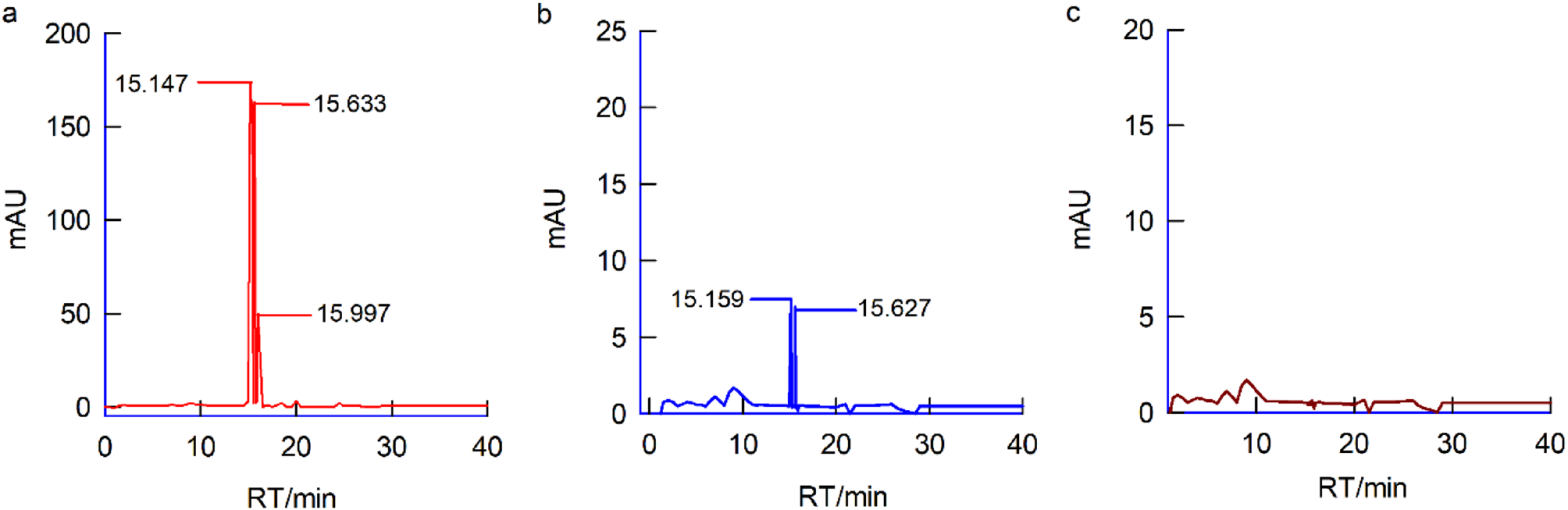
(**a**) HPLC of neem bark extract with ethyl alcohol as solvent having R_t_ = 15.14, 15.63 and 15.99 min, (**b**) HPLC of neem bark extract with water as solvent having R_t_ = 15.15 and 15.62 min, (**c**) HPLC of BCDs with no marked signals.

In Fig. 4a, we present the UV spectrum of BCDs, featuring peaks at λ_max_ = 210 and 274 nm, corresponding to electronic transitions of π–π* (C=C) and n–π* (C=O) energy levels^65–68^. Figure 4b illustrates a typical zeta potential measurement of BCDs, revealing a surface charge peak at − 9.9 eV, indicating a negative surface charge of the BCDs particles. The carbon–oxygen functional groups in BCDs likely exist in a deprotonated structural form. To investigate molecular-level structural changes, we subjected neem bark extracts (ethyl alcohol and water) and BCDs to High-Performance Liquid Chromatography (HPLC) analysis. Specific peaks at retention times, R_t_ = 15.14, 15.63, and 15.99 min with ethyl alcohol (Fig. 5a) and R_t_ = 15.15 and 15.62 min with water solvent (Fig. 5b) were observed in neem bark samples, indicating the presence of various phytochemicals. Remarkably, when BCDs were subjected to HPLC analysis, no significant signals were observed (Fig. 5c).

This observation suggests that the molecular species present in neem bark extracts were transformed into bulk graphene oxide-based BCDs under intense ultrasonication conditions, like the bottom-up approach used for synthesizing graphene quantum dots from carbon-containing molecular systems^55,56,63,64,69^. Detailed instrumental parameters, sample preparation, and HPLC procedures are provided in the supplementary data. The turbidity of BCDs and neem bark extract was determined. The purpose of this study was to examine the formation, dispersion, and stability of BCDs prepared using ultrasonication. Turbidity measurements of BCDs were conducted using a nephelometer, with the calibration procedure provided in the supplementary data. On the first day, the turbidity of BCDs was measured at 425 NTU, and after 35 days, it decreased to 387 NTU. Interestingly, these results indicate that the prepared BCDs were quite stable and well-dispersed over time. In contrast, no turbidity was detected in the neem bark extract. These experimental findings suggest that ultrasonically prepared BCDs remain properly dispersed and stable for several days (see Fig. S2).

### Adsorption of MB on BCDs

The adsorption studies were conducted to evaluate the percentage of removal (Eq. 1)^70,71^, capacity (*Q*_t_) (Eq. 2)^70,71^, equilibrium (*Q*_e_) (Eq. 3)^70,71^, and the efficiency of MB dye removal on BCDs. All the experiment were carried out in 100 ml beaker contain 45 ml of 10 mg L^–1^ MB dye (except the effect of concentration of MB dye) and 5 ml of adsorbents (BCDs). In this context, various solution phase parameters were interrelated, such as the dose of adsorbents (Dilution factor, *D*_f_, ranging from 0 to 10) (Supplementary Table ST1), MB dye concentration (ranging from 1 to 10 mg L^–1^) (Supplementary Table ST2), and experimental conditions like pH (ranging from 3 to 12) (Supplementary Table ST3), added salts (Supplementary Table ST4), real sample matrix (tap water with TDS 620 mg L^–1^) (Supplementary Table ST5), stirring conditions (Supplementary Table ST6), contact time (up to 24 h) (Supplementary Table ST7), and the slow addition of BCDs (0.5 ml added for every 5 intervals) (Supplementary Table ST8) were systematically studied. After completing all procedures, the experiment was left on standby for 24 h to allow the adsorption process to reach completion. The concentration of non-adsorbed material remaining was calculated using the Beer-Lambert law (Eq. 1), while the percentage of removal (Eq. 2), adsorption capacity (Eq. 3), and adsorption equilibrium (Eq. 4) were analysed to investigate the behaviour of BCDs.

1 $$A= \in bc,$$

2 $$\mathrm{Removal \,\,Percentage}= \frac{\left({{\text{C}}}_{{\text{o}}}-{{\text{C}}}_{{\text{t}}}\right) }{{{\text{C}}}_{{\text{o}}}} \times 100,$$

3 $$\mathrm{Adsorption \,\,Capacity }({\text{Qt}})= \frac{\left({{\text{C}}}_{{\text{o}}}-{{\text{C}}}_{{\text{t}}}\right)\mathrm{ V}}{{\text{m}}}$$

4 $$\mathrm{Adsorption \,\,Equilibrium }({\text{Qe}})= \frac{\left({{\text{C}}}_{{\text{o}}}-{{\text{C}}}_{{\text{e}}}\right)\mathrm{ V}}{{\text{m}}}$$

where A is an absorbance measured at 664 nm (which is characterized peak of MB dyes) using UV–Vis spectroscopy, c corresponds to the measure concentration of dyes, $\in$ is the molar absorptivity, and b is the path length, adsorption capacity (Q_t_) and adsorption equilibrium (Q_e_) are expressed in mg g^–1^. C_0_, C_t_, and C_e_ denote the initial concentration of the dye, the concentration of the dye at a specific time, and the quantity of dye adsorbed at equilibrium, respectively. V stands for the volume of the MB dye solution in litres, while *m* represents the weight of the adsorbent, which consists of carbon quantum dots, in grams.

From the collective experimental results, it was optimized that the maximum adsorption capacity and percentage of removal were 605 mg g^–1^ and 64.70%, respectively. The adsorption experiments were conducted ranging from 1 to 14 pH level and the results, revealing that the excellent adsorption efficacy at pH 7, as illustrated in Supplementary Table ST3. The ideal conditions for adsorption were determined to be a *D*_f_ of 10 for BCDs and an MB dye concentration of 10 mg L^–1^ at pH 7. The MB dye adsorption capacity achieved by this method was compared with those reported by other researchers in Table 1^21,47–54^. Although adsorption conditions may vary among different adsorbents, only the effectiveness of various adsorbents was considered for comparison. The results of this study demonstrate that BCDs (with an adsorption capacity of 605 mg g^–1^) outperformed several other adsorbents in terms of adsorption performance. Our findings indicate that prefabricated, cost-effective, and environmentally friendly BCDs have significant potential for adsorbing pollutants such as MB dye from contaminated water.

The observation of the higher sorption capacity of BCDs was attributed to the cooperative effects of (i) the graphitic structure (facilitating π-π interactions), (ii) carbon–oxygen functional groups (which support multiple hydrogen bonding with MB dye), and (iii) anionic surface charge (attracting cationic MB dye).

### Kinetics, isotherm, and thermodynamical studies MB adsorption

The cumulative experimental data obtained under various conditions, including different ratios of BCDs and MB dye as well as varying temperatures, were used to investigate several aspects. This included kinetics, examined through pseudo-first order (as shown in Eq. (5), Supporting Information Fig. S3) and pseudo-second-order (as shown in Fig. 7a and Eq. (6)) analyses^72–75^. Additionally, the study encompassed isotherm analysis using Langmuir (Eqs. 7 and 8) and Freundlich isotherm (Eq. 9), with their respective parameters shown in Fig. S4^72–75^. Lastly, thermodynamic parameters, such as ΔG, were determined using Eqs. 10 and 11, and the results can be found in the Supplementary Table ST9. For comparison purpose, the coefficients of kinetics (pseudo-first order and pseudo-second-order), isotherm (Langmuir and Freundlich), and thermodynamic studies (ΔG) are provided in the Table 2^72–75^.

5 $${\text{ln}}\left({{\text{Q}}}_{{\text{e}}}-{{\text{Q}}}_{{\text{t}}}\right)={\text{ln}}\left({{\text{Q}}}_{{\text{e}}}\right)- {{\text{k}}}_{1}{\text{t}},$$

6 $$\frac{{\text{t}}}{{{\text{Q}}}_{{\text{t}}}}= \frac{1}{{{\text{k}}}_{2}{{\text{Q}}}_{{\text{e}}}^{2}}+ \frac{{\text{t}}}{{{\text{Q}}}_{{\text{e}}}},$$

7 $$\frac{{{\text{C}}}_{{\text{e}}}}{{{\text{Q}}}_{{\text{e}}}}= \frac{1}{{{\text{Q}}}_{{\text{max}}} {{\text{K}}}_{{\text{L}}}} + \frac{{{\text{C}}}_{{\text{e}}}}{{{\text{Q}}}_{{\text{max}}}},$$

8 $${{\text{R}}}_{{\text{L}}}=\frac{1}{1+ {{\text{K}}}_{{\text{L}}}{{\text{C}}}_{{\text{o}}}},$$

9 $${{\text{lnQ}}}_{{\text{e}}} ={{\text{lnk}}}_{{\text{f}}} + \frac{1}{{\text{n}}}{{\text{lnC}}}_{{\text{e}}},$$

10 $$\Delta {\text{G}}= -\mathrm{RTln }{{\text{K}}}_{{\text{c}}},$$

11 $${{\text{K}}}_{{\text{c}}}=\frac{{{\text{Q}}}_{{\text{e}}}}{{{\text{C}}}_{{\text{e}}}},$$

where k_1_ (min^–1^) is the rate constant for the pseudo first-order (PFO) model, k_2_ (g mg^–1^ min^–1^) is the rate constant for the pseudo second-order (PSO) model, Q_max_ (mg g^–1^) is the Langmuir constant related to adsorption capacity, K_L_ (L mg^–1^) is the Langmuir constant, R_L_ is the feasibility of the adsorbent, k_f_ (mg g^–1^) is the Freundlich constant related to adsorption energy, K_c_ is the thermodynamic constant, R is the universal gas constant (8.314 J mol^–1^ K^–1^), T is the absolute temperature in Kelvin (K), and ΔG is the Gibbs free energy change (J mol^–1^)^72–75^.

**Table 2:** Coefficients of kinetics, isotherm, and thermodynamic studies for sorption of MB dye on BCDs.

Kinetic and Isotherm models	Coefficients Q_max_ = 605 mg g^–1^ (experimental)	Concentration (mg L^–1^)				
		10	6		2	1
Pseudo first order	K_1_ (min^–1^)	− 0.005	− 0.0275		− 0.0186	0.0145
	R^2^	0.9796	0.8550		0.9743	0.5862
Pseudo second order	K_2_ (g mg^-1^ min^–1^)	6.71 × 10^–4^	2.17 × 10^–9^		2.05 × 10^–3^	− 0.83 × 10^–2^
	R^2^	0.9963	0.9985		0.9962	0.9980
	Q_max_ (mg g^–1^)	333.33	384.61		128.20	34.72
Feasibility of the adsorbent	R_L_	0.4399	0.5669		0.7970	0.8870
Langmuir isotherm	K_L_ (L mg^–1^)			0.1273		
	Q_max_ (mg g^–1^)			714.28		
	R^2^			0.9999		
Freundlich isotherm	K_f_ (mg g^–1^)			38.822		
	1/n			0.7082		
	n			1.4120		
	R^2^			0.9748		
Thermodynamic parameter (ΔG) (J mol^-1^)	− 10,335.1 (at 305 K)					
	− 5931.6 (at 315 K)					
	− 5746.98 (at 325 K)					
	− 5578.38 (at 335 K)					

#### Kinetic studies

The pseudo-first-order and pseudo-second-order adsorption kinetics are typically plotted against time and adsorption capacity (Q_t_). Based on the linear correlation (R2) data, the second-order kinetics experiments showed better linearity than that of the first-order kinetics. This result suggests that the sorption process operates via an electrostatic mechanism, where the MB dye and the functional groups on BCDs share charges^74^.

#### Isotherm studies

The Langmuir isotherm, which was based on the postulate that homogeneous adsorption occurs with uniformly distributed active sites, was thought to be the most consistent of all the isotherms. A linear model for the Langmuir isotherm was plotted between C_e_ and C_e_/Q_e_. Based on the plot results, the R^2^ value is equal to 0.9999, and the maximum theoretical adsorption capacity (Q_max_) was found to be 714 mg g^–1^; however, this study achieved 605 mg g^–1^ using BCDs. The theoretical and experimental data are well associated with each other, emphasizing the excellent compatibility of the Langmuir model with the MB dye adsorption on BCDs. A dimensionless separation factor (R_L_) has been calculated via the Langmuir constant (K_L_), with R_L_ values ranging from 0 to 1 (0.4399, 0.5669, 0.7970, and 0.8870). The outcome of the R_L_ constant indicates that the adsorption of MB dye on BCDs was favourable.

The Freundlich isotherm describes heterogeneous adsorption (multilayer mechanism) as an empirical equation, and a plot is drawn between ln Q_e_ and ln C_e._ For this model, the R^2^ value is 0.9747, indicating that the Freundlich isotherm doesn’t fit the experimental data as well as the Langmuir isotherm. However, the empirical parameters of the Freundlich isotherm are still considered. The adsorption intensity (n) reveals that the adsorption process is physical and favourable (n is greater than 1).

#### Thermodynamic study

To calculate thermodynamic parameters (Gibbs free energy, ΔG), the adsorption studies were carried out at four different temperatures (305 K, 315 K, 325 K, and 335 K) (see Supplementary Table ST9 and Table 2). It was noted that the Gibbs free energy (ΔG) at all temperatures explored was negative, indicating that the adsorption of MB dye on BCDs was spontaneous and feasible^72–75^.

## Comparison of BCDs with conventional materials for MB adsorption

The adsorption efficiency of BCDs was compared to that of well-known adsorbents such as activated charcoal, neutral alumina, and basic alumina. In batch experiments, we employed four containers, each filled with a solution containing 10 mg L^–1^ of MB dye. Additionally, different adsorbents were introduced into each container: the first contained 1.4 g of BCDs, the second held 1.4 g of activated charcoal, the third contained 1.4 g of basic alumina, and the fourth housed 1.4 g of neutral alumina. Figure 7b & c displays the typical UV–Vis response of BCDs, charcoal, and MB dye under variable experimental conditions. It is evident that the BCDs-based dye removal reaction resulted in the complete disappearance of the MB dye signal, whereas other control experiments produced trace amounts and marked signals of MB dye (additional time). The real-time dye removal experimental results were visualized and presented in Fig. S5. It is obvious that the solution treated with BCDs resulted in a clear solution of the MB dye, in contrast to the turbid appearance in the other control experiments, highlighting the superior performance of BCDs.

## Mechanism of adsorption and regeneration of MB dye by BCDs

**Figure 6:**
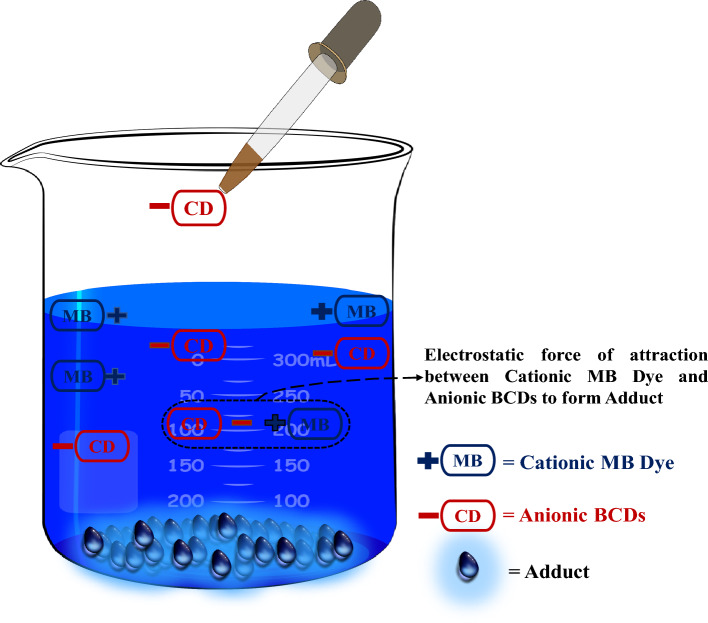
Mechanism of adsorption.

The plausible mechanism for the adsorption of cationic MB dye on anionic BCDs has been revealed based on the structure, kinetics, isotherms, thermodynamics, and zeta potential results. The electrostatic force of attraction between the anionic charge of BCDs (-ve) and the cationic charge of MB dye (+ ve) drove the robust adsorption/interaction process, resulting in the formation of an adduct between them (Fig. 6).

**Figure 7:**
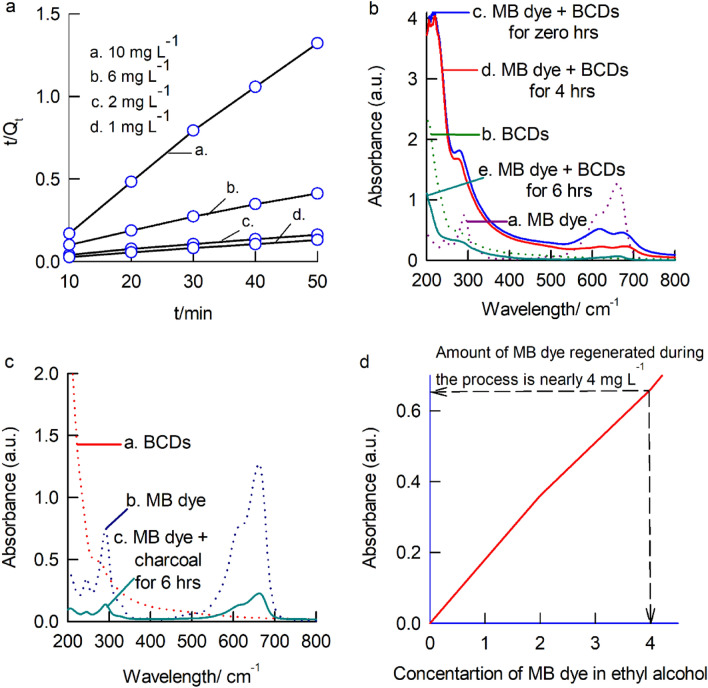
(**a**) The kinetic plot of pseudo second order which shows better linear correlation for all concentration from 1 to 10 mg L^–1^, (**b**) UV–Vis response of BCDs and MB dye at variable experimental conditions, (**c**) UV–Vis response of charcoal and MB dye at variable experimental conditions, (**d**) Regeneration of MB dye using ethyl alcohol.

In addition to dye removal based on the adduct, successful regeneration of MB from the reaction mixture was achieved in this work. The recovery study was conducted using the following procedure: the adduct was centrifuged at 5000 rpm for 30 min and then filtered through a 0.22 µm cellulose membrane. The solid adduct was mixed with 100 ml of ethyl alcohol, gently stirred, and left at room temperature (T = 25 ± 2 °C) for 5 min. The clear ethanolic solution containing MB dye was separated by simple filtration (Fig. S6). According to the adsorption data, 64% of the MB dye was successfully recovered, as shown in Fig. 7d.

**Figure 8:**
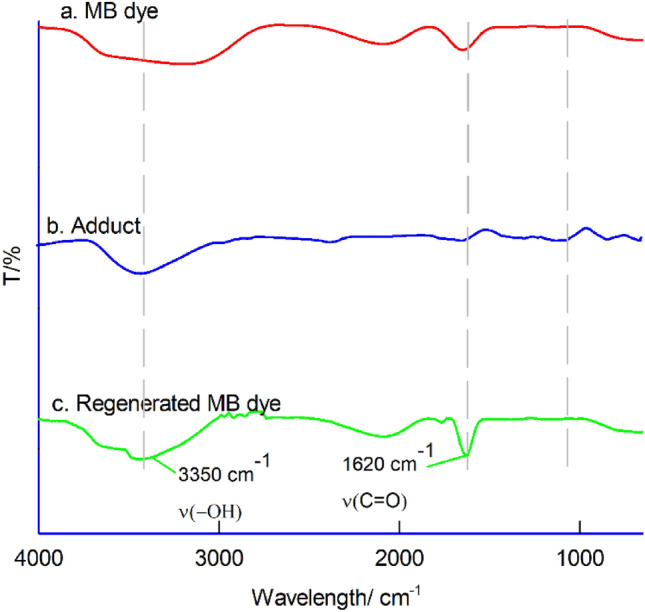
The IR spectrum of a. MB dye, b. adduct, c. regenerated MB dye.

To confirm the rejuvenation of MB dye from the adduct, IR spectra of MB dye, the adduct, and the regenerated MB dye were analysed. The spectral results of the regenerated MB dye (from the adduct) match with the IR spectrum of native MB dye, confirming the successful rejuvenation of MB dye from the adduct, as depicted in Fig. 8.

The BCDs prepared in this study exhibit a remarkable adsorption capacity of 605 mg g^–1^ and achieve a removal percentage of 64.7% for MB dye from aqueous solutions. The adsorption process demonstrates a negative ΔG value, follows pseudo-second-order kinetics, and conforms to the Langmuir isotherm. Effective extraction of the adsorbed MB dye is achieved using ethyl alcohol as a solvent, with a recovery rate of 64% based on the adsorption data. The residual BCDs from this process hold potential for utilization as green manure, particularly if scaled up for industrial applications. The entire procedure is characterized by its economic viability, ecological benignity, and adherence to the principles of green chemistry. Comparative analysis with previously reported methods reveals that this approach is more sustainable and cost-effective, ensuring complete removal of cationic dye without the use or production of toxic substances, thus avoiding decomposition.

## Conclusion

Ultrasonication is a conventional technique in several research areas of nanoparticle synthesis. However, it's a fact that the process itself can lead to the formation of quantum dots, which remains unexplored. In this study, we attempted to synthesize graphene oxide layered BCDs from neem bark biomass using ultrasonication for 8 h. The carbon–oxygen-functionalized graphene oxide layered BCDs were characterized using UV–Vis, FTIR, Raman, XRD, HRTEM, FESEM, EDAX, and Zeta potential. In comparison to many sorbents mentioned in the literature, the adsorption capacity and removal percentage were found to be 605 mg g^–1^ and 64.7% respectively, which was quite high. The BCDs was used as a liquid-phase adsorbent for the regenerative adsorption of the environmentally polluting cationic dye, such as MB dye, via an in-situ precipitation reaction (BCDs-MB adduct formation; electrostatic mechanism). Subsequently, it was used for regenerating MB dye was regenerated using a solvent exchange method. Consistent with the sorption process, we successfully retained 64% of the MB dye during the regeneration process. The Langmuir isotherm and pseudo-second-order kinetics demonstrated significant linearity among all systems. The computed thermodynamic parameter, Gibbs free energy (ΔG), was found to be negative. The outcomes of the kinetic, isotherm, and thermodynamic models suggest that the adsorption process was homogeneous, exothermic, spontaneous, and physisorption. Using this simple procedure, we were able to extract a valuable cationic surfactant from the aqueous solution. An extension of this study will likely lead to substantial developments and that will support large-scale industrial applications.

### Supplementary Information

Supplementary Information.

## Data Availability

All data generated or analysed data for experimental part during this study are included in this published article and its supplementary information file.

## Abbreviations

BCDs: Biomass carbon dots
DBCDs: Dried biomass carbon dots
MB Dye: Methylene blue dye
Df: Dilution factor

## References

[1] El-Shamy AG (2020). Synthesis of new magnesium peroxide (MgO_2_) nano-rods for pollutant dye removal and antibacterial applications. Mater. Chem. Phys..

[2] El-Shamy AG, Zayied HSS (2020). New polyvinyl alcohol/carbon quantum dots (PVA/CQDs) nanocomposite films: structural, optical and catalysis properties. Synth. Met..

[3] Bibi M, Ajmal M, Naseer F, Farooqi ZH, Siddiq M (2017). Preparation of magnetic microgels for catalytic reduction of 4-nitrophenol and removal of methylene blue from aqueous medium. Int. J. Environ. Sci. Technol..

[4] Mohammed MA, Shitu A, Ibrahim A (2014). Removal of methylene blue using low cost adsorbent: A review. Res. J. Chem. Sci..

[5] Ren L, Tang Z, Du J, Chen L, Qiang T (2021). Recyclable polyurethane foam loaded with carboxymethyl chitosan for adsorption of methylene blue. J. Hazard Mater..

[6] Temel F, Turkyilmaz M, Kucukcongar S (2020). Removal of methylene blue from aqueous solutions by silica gel supported calix [4] arene cage: Investigation of adsorption properties. Eur. Polym. J..

[7] Verma AK, Dash RR, Bhunia P (2012). A review on chemical coagulation/flocculation technologies for removal of colour from textile wastewaters. J. Environ. Manag..

[8] Yu S, Liu M, Ma M, Qi M, Lü Z, Gao C (2010). Impacts of membrane properties on reactive dye removal from dye/salt mixtures by asymmetric cellulose acetate and composite polyamide nanofiltration membranes. J. Membr. Sci..

[9] Asghar A, Abdul Raman AA, Wan Daud WMA (2015). Advanced oxidation processes for in-situ production of hydrogen peroxide/hydroxyl radical for textile wastewater treatment: A review. J. Clean. Prod..

[10] Kim I, Tanaka H (2010). Use of ozone-based processes for the removal of pharmaceuticals detected in a wastewater treatment plant. Water Environ. Res..

[11] Kordouli E, Bourikas K, Lycourghiotis A, Kordulis C (2015). The mechanism of azo-dyes Adsorption on the titanium dioxide surface and their photocatalytic degradation over samples with various anatase/rutile ratios. Catal. Today.

[12] Ali H, Ahmad W, Haq T (2009). Decolorization and degradation of malachite green by *Aspergillus*
*flavus* and *Alternaria*
*solani*. Afr. J. Biotechnol..

[13] Cao J, Zhang J, Zhu Y, Wang S, Wang X, Lv K (2018). Novel polymer material for efficiently removing methylene blue, Cu (II) and emulsified oil droplets from water simultaneously. Polymers.

[14] Erabee IK, Ahsan A, Jose B, Aziz MMA, Ng AWN, Idrus S, Daud NNN (2018). Adsorptive treatment of landfill leachate using activated carbon modified with three different methods. KSCE J. Civ. Eng..

[15] Hamad HN, Idrus S (2022). Recent developments in the application of bio-waste-derived adsorbents for the removal of methylene blue from wastewater: A review. Polymers.

[16] Karaca S, Gürses A, Açıkyıldız M, Ejder MK (2008). Adsorption of cationic dye from aqueous solutions by activated carbon. Microporous Mesoporous Mater..

[17] Sadegh H, Ali GAM, Gupta VK, Makhlouf ASH (2017). The role of nanomaterials as effective adsorbents and their applications in wastewater treatment. JNSC.

[18] Vakili M, Rafatullah M, Salamatinia B, Abdullah AZ, Ibrahim MH, Tan KB, Gholami Z, Amouzgar P (2014). Application of chitosan and its derivatives as adsorbents for dye removal from water and wastewater: A review. Carbohydr. Polym..

[19] Nazim M, Khan AAP, Asiri AM, Kim JH (2021). Exploring rapid photocatalytic degradation of organic pollutants with porous CuO nanosheets: Synthesis, dye removal, and kinetic studies at room temperature. ACS Omega.

[20] Vasiljevic ZZ, Dojcinovic MP, Vujancevic JD, Jankovic-Castvan I, Ognjanovic M, Tadic NB, Stojadinovic S, Brankovic GO, Nikolic MV (2020). Photocatalytic degradation of methylene blue under natural sunlight using iron titanate nanoparticles prepared by a modified sol-gel method. R. Soc. Open Sci..

[21] Li Y, Wang S, Shen Z, Li X, Zhou Q, Sun Y, Wang T, Liu Y, Gao Q (2020). Gradient adsorption of methylene blue and crystal violet onto compound microporous silica from aqueous medium. ACS Omega.

[22] Rovani S, Santos JJ, Corio P, Fungaro DA (2018). Highly pure silica nanoparticles with high adsorption capacity obtained from sugarcane waste ash. ACS Omega.

[23] Iqbal Z, Tanweer MS, Alam M (2023). Reduced graphene oxide-modified spinel cobalt ferrite nanocomposite: Synthesis, characterization, and its superior adsorption performance for dyes and heavy metals. ACS Omega.

[24] Li P, Zhao T, Zhao Z, Tang H, Feng W, Zhang Z (2023). Biochar derived from chinese herb medicine residues for rhodamine B dye adsorption. ACS Omega.

[25] Chen Q, Wang H, Tang X, Ba Z, Zhao X, Wang Y, Deng H (2021). One-step synthesis of carbon quantum dot-carbon nanotube composites on waste eggshell-derived catalysts for enhanced adsorption of methylene blue. J. Environ. Chem. Eng..

[26] Kurian M, Paul A (2021). Recent trends in the use of green sources for carbon dot synthesis–a short review. Carbon Trends.

[27] Atabaev TS (2018). Doped carbon dots for sensing and bioimaging applications: A minireview. Nanomaterials.

[28] Miao S, Liang K, Zhu J, Yang B, Zhao D, Kong B (2020). Hetero-atom-doped carbon dots: doping strategies, properties and applications. Nano Today.

[29] Zhang Z, Yi G, Li P, Zhang X, Fan H, Zhang Y, Wang X, Zhang CA (2020). Minireview on doped carbon dots for photocatalytic and electrocatalytic applications. Nanoscale.

[30] Tyagi A, Tripathi KM, Singh N, Choudhary S, Gupta RK (2016). Green synthesis of carbon quantum dots from lemon peel waste: Applications in sensing and photocatalysis. RSC Adv..

[31] Liam JD, Anh NP, Piergiorgio G (2021). Critical overview on the green synthesis of carbon quantum dots and their application for cancer therapy. Environ. Sci. Nano.

[32] Xia C, Zhu S, Feng T, Yang M, Yang B (2019). Evolution and synthesis of carbon dots: From carbon dots to carbonized polymer dots. Adv. Sci..

[33] de Medeiros TV, Manioudakis J, Noun F, Macairan JR, Victoria F, Naccache R (2019). Microwave-assisted synthesis of carbon dots and their applications. J. Mater. Chem. C.

[34] Desai ML, Jha S, Basu H, Singhal RK, Park TJ, Kailasa SK (2019). Acid oxidation of muskmelon fruit for the fabrication of carbon dots with specific emission colours for recognition of Hg^2+^ ions and cell imaging. ACS Omega.

[35] Wu FS, Yang MQ, Zhang H, Zhu SZ, Zhu XJ, Wang K (2018). Facile synthesis of sulfur-doped carbon quantum dots from vitamin B_1_ for highly selective detection of Fe^3+^ ion. Opt. Mater..

[36] Facure MHM, Schneider R, Mercanta LA, Correa DS (2022). Rational hydrothermal synthesis of graphene quantum dots with optimized luminescent properties for sensing applications. Mater. Today Chem..

[37] Muthukumaran M, Basha ZW, Venkatachalam K, Rasheeth A (2022). A new chemically modified carbon paste electrode derived from aloe vera xanthate nanoparticles to detect mercury ions. Electroanalysis.

[38] Balakrishnan T, Aug WL, Mahmoudi E, Mohammad AW, Sambudi NS (2022). Formation mechanism and application potential of carbon dots synthesized from palm kernel shell via microwave assisted method. Carbon Resour..

[39] Lee YS, Hu CC, Chiu TC (2022). Electrochemical synthesis of fluorescent carbon dots for the selective detection of chlortetracycline. J. Environ. Chem. Eng..

[40] Manju K, Anju P (2021). Recent trends in the use of green sources for carbon dot synthesis–a short review. Carbon Trends.

[41] Zhu G, Fang H, Xiao Y (2020). The application of fluorescence spectroscopy for the investigation of dye degradation by chemical oxidation. J. Fluoresc..

[42] Lee SY, Kang D, Jeong S, Do HT, Kim JH (2020). Photocatalytic degradation of rhodamine b dye by TiO_2_ and gold nanoparticles supported on a floating porous polydimethylsiloxane sponge under ultraviolet and visible light irradiation. ACS Omega.

[43] Yahya R, Shah A, Kokab T, Ullah N, Hakeem MK, Hayat M, Haleem A, Shah I (2022). Electrochemical sensor for detection and degradation studies of ethyl violet dye. ACS Omega.

[44] Verma R, Singh J, Samdarshi SK, Srivastava A (2022). Phase modulation kinetics in TiO_2_ by manipulating pH: A dynamic of photoactivity at different combination of phase and pH. J. Alloys Compd..

[45] Verma R, Samdarshi SK, Sagar K, Konwar BK (2017). Nanostructured bi-phasic TiO_2_ nanoparticles grown on reduced graphene oxide with high visible light photocatalytic detoxification. Mater. Chem. Phys..

[46] Khanal S (2021). Qualitative and quantitative phytochemical screening of azadirachtaindicajuss. Plant Parts. Int. J. Appl. Sci. Biotechnol..

[47] Li Y, Zimmerman AR, He F, Chen J, Han L, Chen H, Hu X, Gao B (2020). Solvent-free synthesis of magnetic biochar and activated carbon through ball-mill extrusion with Fe_3_O_4_ nanoparticles for enhancing adsorption of methylene blue. Sci. Total Environ..

[48] Bentahar S, Dbik A, El Khomri M, El Messaoudi N, Lacherai A (2018). Removal of a cationic dye from aqueous solution by natural clay. Groundw. Sustain. Dev..

[49] Liu XJ, Li MF, Singh SK (2021). Manganese-modified lignin biochar as adsorbent for removal of methylene blue. J. Mater. Res. Technol..

[50] Termeh T, Nazanin H, Mohammad HM, Zahra E (2021). N, S doped carbon quantum dots inside mesoporous silica for effective adsorption of methylene blue dye. SN Appl. Sci..

[51] Saleh AT, Al-Ruwayshid Saad H, SarÄ A, Tuzen M (2020). Synthesis of silica nanoparticles grafted with copolymer of acrylic acrylamide for ultra-removal of methylene blue from aquatic solutions. Eur. Polym. J..

[52] Adeyi AA, Md Jamil SNA, Abdullah LC, Choong TSY, Lau KL, Abdullah M (2019). Adsorptive removal of methylene blue from aquatic environments using thiourea modified poly (acrylonitrile-co-acrylic acid). Materials.

[53] Zhang F, Yin X, Zhang W, Ji Y (2016). Optimizing decolonization of methyl blue solution by two magnetic hydroxyapatite nanorods. J. Taiwan Inst. Chem. Eng..

[54] Peres EC, Slaviero JC, Cunha AM, Hosseini-Bandegharaei A, Dotto GL (2017). Microwave synthesis of silica nanoparticles and its application for methylene blue adsorption. J. Environ. Chem. Eng..

[55] Chen BB, Liu ZX, Deng WC, Zhan L, Liu ML, Huang CZ (2016). A large-scale synthesis of photoluminescent carbon quantum dots: A self-exothermic reaction driving the formation of the nanocrystalline core at room temperature. Green Chem..

[56] Ding Z, Li F, Wen J, Wang X, Sun R (2018). Gram-scale synthesis of single-crystalline graphene quantum dots derived from lignin biomass. Green Chem..

[57] Dong Y, Pang H, Yang HB, Guo C, Shao J, Chi Y, Li CM, Yu T (2013). Carbon-based dots Co-doped with nitrogen and sulfur for high quantum yield and excitation-independent emission. Angew. Chem..

[58] Selvaraju N, Ganesh PS, Palrasu V, Venugopal G, Mariappan V (2022). Evaluation of antimicrobial and antibiofilm activity of citrus media fruit juice-based carbon dots against *Pseudomonas*
*aeruginosa*. ACS Omega.

[59] Wu M, Zhan J, Geng B, He P, Wu K, Wang L, Xu G, Li Z, Yin L, Pan D (2017). Scalable synthesis of organic-soluble carbon quantum dots: Superior optical properties in solvents, solids, and LEDs. Nanoscale.

[60] Kwon W, Do S, Kim J-H, Seok Jeong M, Rhee S-W (2015). Control of photoluminescence of carbon nanodots via surface functionalization using para-substituted anilines. Sci. Rep..

[61] Mishra S, Das K, Chatterjee S, Sahoo P, Kundu A, Pal M, Bhaumik A, Ghous CK (2023). Facile and green synthesis of novel fluorescent carbon quantum dots and their silver heterostructure: An in vitro anticancer activity and imaging on colorectal carcinoma. ACS Omega.

[62] López-Díaz D, López Holgado M, Fierro JLG, Velázquez MM (2017). The evolution of the Raman spectrum with the chemical composition of graphene oxide. J. Phys. Chem. C.

[63] Park YR, Jeong HY, Seo YS, Choi WK, Hong YJ (2017). Quantum-dot light-emitting diodes with nitrogen-doped carbon nanodot hole transport and electronic energy transfer layer. Sci. Rep..

[64] Hola K (2017). Graphitic nitrogen triggers red fluorescence in carbon dots. ACS Nano.

[65] De B, Karak N (2013). A green and facile approach for the synthesis of water-soluble fluorescent carbon dots from banana juice. RSC Adv..

[66] Ma Z, Ming H, Huang H, Liu Y, Kang Z (2012). One-step ultrasonic synthesis of fluorescent N-doped carbon dots from glucose and their visible-light sensitive photocatalytic ability. N. J. Chem..

[67] Roy P, Chen PC, Periasamy AP, Chen YN, Chang HT (2015). Photoluminescent carbon nanodots: Synthesis, physicochemical properties and analytical applications. Mater. Today.

[68] Shaikh AF, Tamboli MS, Patil RH, Bhan A, Ambekar JD, Kale BB (2019). Bioinspired carbon quantum dots: An antibiofilm agents. J. Nanosci. Nanotechnol..

[69] Preethi M, Murugan R, Viswanathan C, Ponpandian N (2022). Potato starch derived N-doped carbon quantum dots as a fluorescent sensing tool for ascorbic acid. J. Photochem..

[70] Barbosa Jde (2019). Eudragit E100/poly (ethylene oxide) electrospun fibers for DNA removal from aqueous solution. J. Appl. Polym. Sci..

[71] Mall ID, Srivastava VC, Agarwal NK (2006). Removal of orange-G and methyl violet dyes by adsorption onto bagasse fly ash kinetic study and equilibrium isotherm analyses. Dyes Pigm..

[72] Kuang Y, Zhang X, Zhou S (2022). Adsorption of methylene blue in water onto activated carbon by surfactant modification. Water.

[73] Sterenzon E, Vadivel VK, Gerchman Y, Luxbacher T, Narayanan R, Mamane H (2022). Effective removal of acid dye in synthetic and silk dyeing effluent: Isotherm and kinetic studies. ACS Omega.

[74] Haq A (2022). Biosorption of metribuzin pesticide by cucumber (*Cucumis*
*sativus*) peels-zinc oxide nanoparticles composite. Sci. Rep..

[75] Khan MI, Shanableh A, Elboughdiri N, Lashari MH, Manzoor S, Shahida S, Farooq N, Bouazzi Y, Rejeb S, Elleuch Z, Kriaa K, Rehman A (2022). Adsorption of methyl orange from an aqueous solution onto a BPPO-based anion exchange membrane. ACS Omega.

