# Supplementary material for: Neem biomass derived carbon quantum dots synthesized via one step ultrasonification method for ecofriendly methylene blue dye removal
Source: Sci Rep. 2024 Apr 27;14:9706. doi: 10.1038/s41598-024-59483-9 (PMC11055862; doi:10.1038/s41598-024-59483-9)
Supplement: Supplementary file 1 — Supplementary Information. [file 41598_2024_59483_MOESM1_ESM.pdf]

Supporting Information

Neem Biomass Derived Carbon Quantum Dots Synthesized via One Step Ultrasonification Method for Ecofriendly Methylene Blue Dye Removal

Z. Waseem Basha[a], S. Muniraj[a] and Annamalai Senthil Kumar[b*]*

[a] P.G. & Research Department of Chemistry, RKM Vivekananda College (Autonomous), Mylapore, Chennai-600004.

[b] Nano and Bioelectrochemistry Research Laboratory, Carbon dioxide and Green Technology Research Centre and Department of Chemistry, School of Advance Science, Vellore, Institute of Technology University, Vellore- 632 014, Tamil Nadu, India.

Corresponding author's email:smuniraj@rkmvc.ac.in (muniraj)
andaskumarchem@yahoo.com (a.senthilkumar)

Experimental Section

1. Characterizations or analytical methods

The morphology and topology of the BCDs were investigated with the help of the HRTEM using a JEOL-JEM-2100 plus devise. The structure and composition of BCDs was analysed using Aeries, Panalytical powder X-ray diffraction instrument. Fourier transform infrared (FTIR) spectroscopy was examine using a SHIMADZU, IRTRACER 100 spectrophotometer. The photo luminous properties of BCDs were studied utilising UV- Visible spectroscopy, which is a very simple and efficient tool using SHIMADZU UV-3600 plus instrument.

2. Instrumental parameters of high-performance liquid chromatography

2.1 Instrument and its parameters

A comparative HPLC analysis of Neem Bark extracts and BCDs is executed to evaluate compound changes using a Shimadzu Prominence HPLC pump, SPD-M10 DAD detector, CTO-10 oven, and 10 Avp auto sampler. The analysis was performed on a Phenomenex Luna C_{18} Column with a mobile phase of Water: Methanol (30: 70 v/v) and eluted at 35 °C (250 mm x 4.6 mm i.d., 5 m particle size). The detection wavelength was 274 nm, and the flow rate was 1 ml min^{-1}.

2.2 Sample preparation

Two different neem bark extract from that is one from water and another from ethyl alcohol were prepared: neem bark powder 4 g was dispersed in 100 ml of double-distilled water for 24 hr along with an occasionally stirred. The same process was used to make ethyl alcohol extract.

2.3 Procedure

The aqueous solution of BCDs, water and ethyl alcohol neem bark extracts have been injected in to a C_{18} column and eluted at 35 ^{0}C. For all three analyses, a mobile phase consists of water; methanol (30:70) has been used at the flow rate of 1 ml min^{-1} and detected at the wavelength of 274 nm.

2.3.1.Regeneration of MB dye

It has been imperative to regenerate MB dye from the adduct for economic and environmental reasons. In the current research, ethyl alcohol was utilized to conduct the desorption of MB dye from the adduct (solvent exchange method).

2.3.2.Procedure for the calibration of nephelometer

1 g of hydrazine sulphate ($(NH_2)_2$. H_2SO_4) in distilled water and dilutes to 100 ml in a volumetric flask which is consider as a solution I. 10 g of hexamethylenetetramine ($(CH_2)_6N_4$) in distilled water and dilutes to 100 ml in a volumetric flask which is denoted as solution II. In a flask, 5.0 ml of solution I and 5 ml of solution II and allow to stand for 24 hr at 25 ± 3 ^{o}C which result in the white colour suspension having 4000 nephelometric turbidity units (NTU). The white colour suspension is stored in an amber glass bottle which is stable up to 1 year. The 4000 NTU white colour suspended stock solution was diluted with distilled water to prepare diluted standard just before the use and discard after use. Using the white colour suspension (4000 NTU), the nephelometer was calibrated according to the manufacturer’s operating instructions.

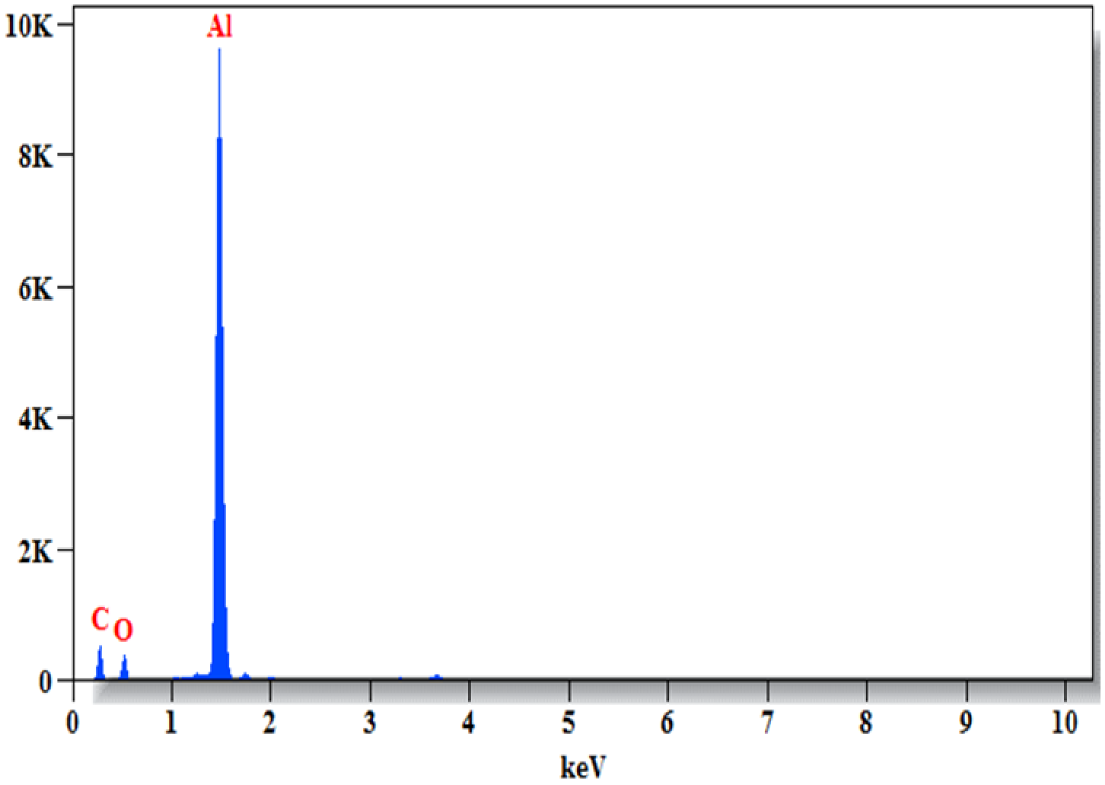

Element	Net Counts	Weight %	Atom %	Formula
C	3074	64.16	70.44	C
O	2419	35.85	29.55	O
Total		100.00	100.00	

Fig. S1 EDAX of BCDs having carbon and hydrogen as major components.

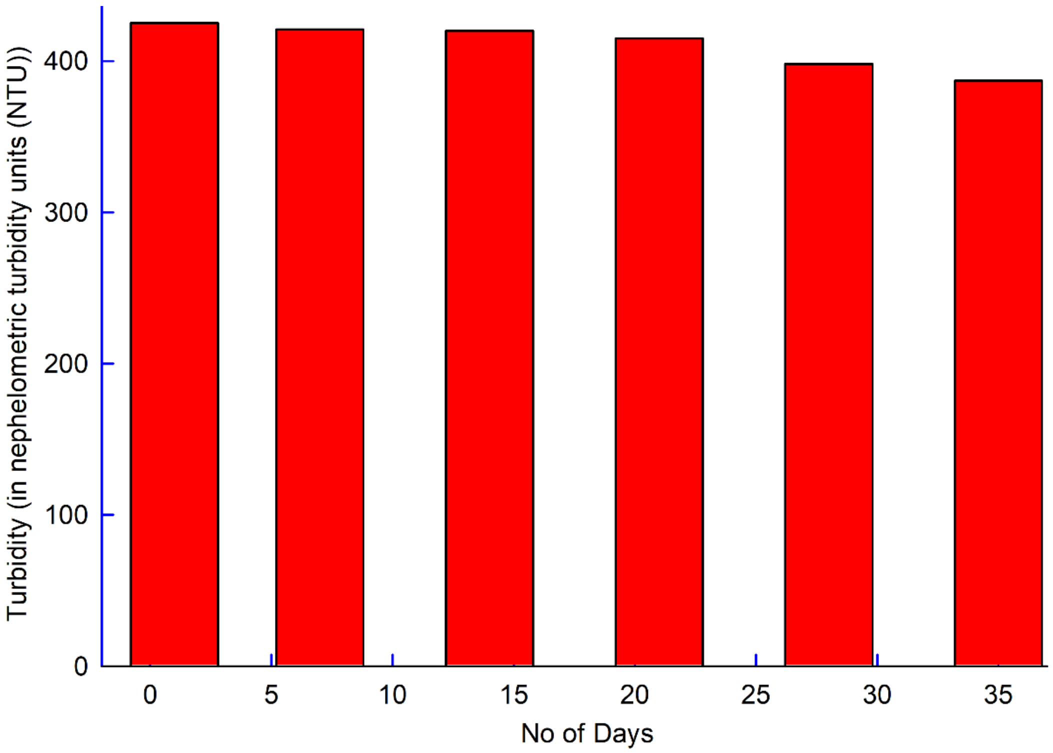

Fig. S2 Formational change, proper dispersion, stability of BCDs using nephelometer.

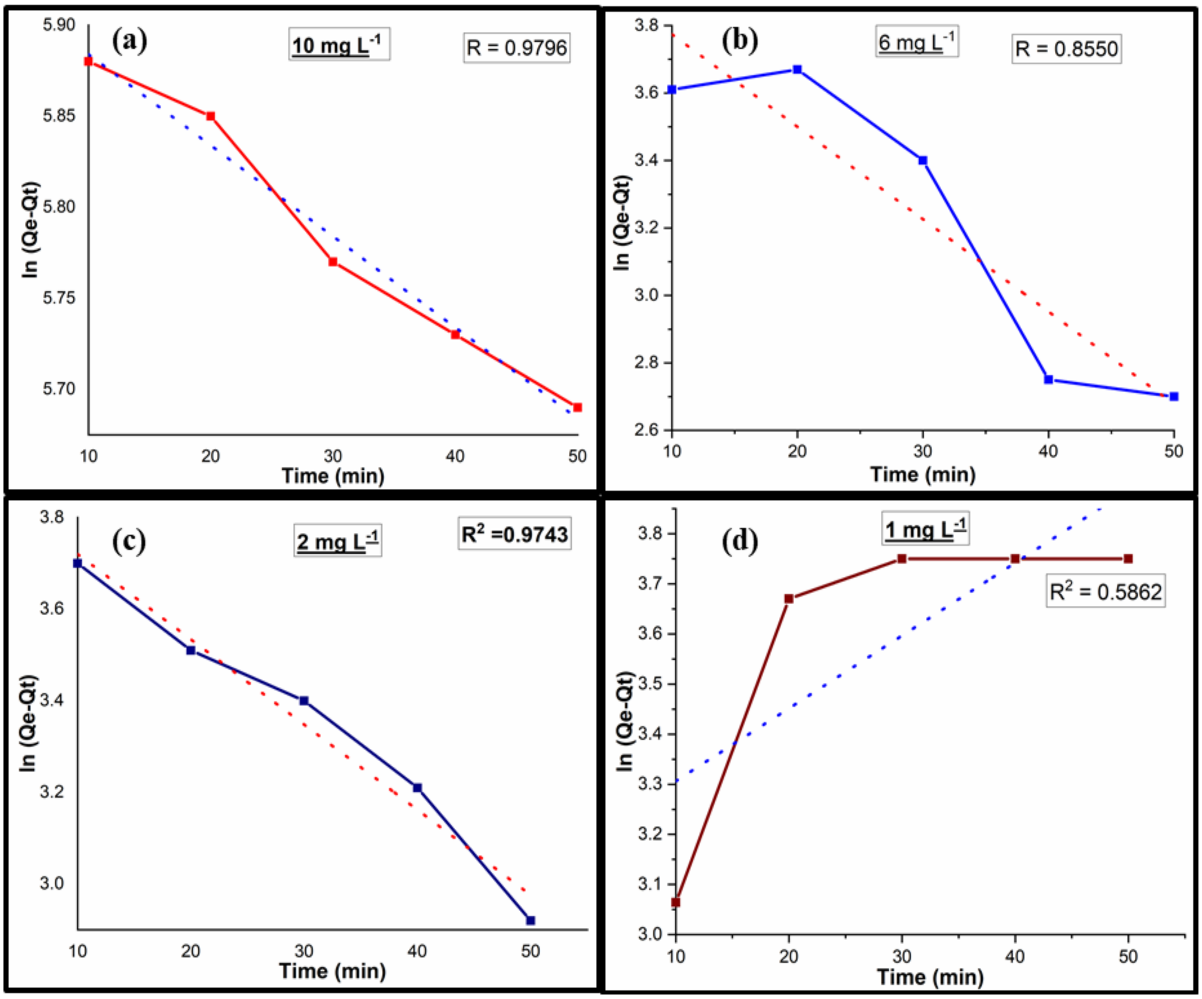

Fig. S3 pseudo first order model for different concentration **(a)** 10 mg. L^{-1} **(b)** 6 mg. L^{-1} **(c)** 2 mg. L^{-1} **(d)** 1 mg. L^{-1}

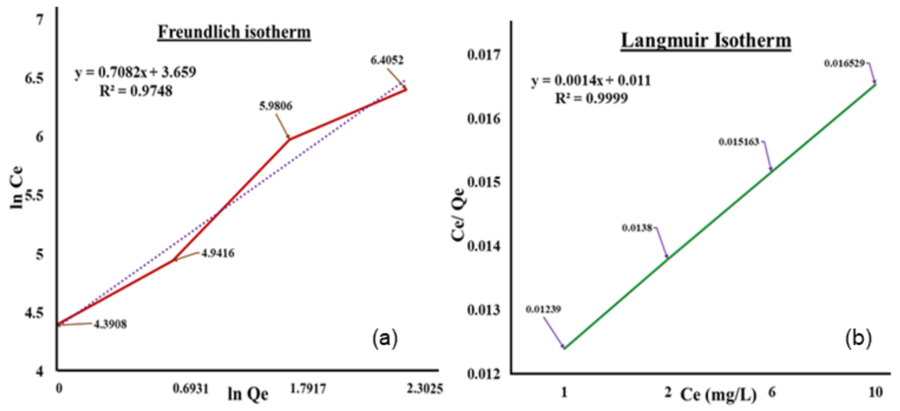

K_f	38.82 mg. g^{-1}
n	1.4020
Q_{max}	714.28 mg. g^{-1}
K_L	0.1273 L. mg^{-1}
R_L	0.4399<br>0.5669<br>0.7970<br>0.8870

(c)

Fig. S4 (a) Freundlich isotherm of adsorption of MB dye on BCDs (b) Langmuir isotherm of adsorption of MB dye on BCDs (c) constant values of Isotherm.

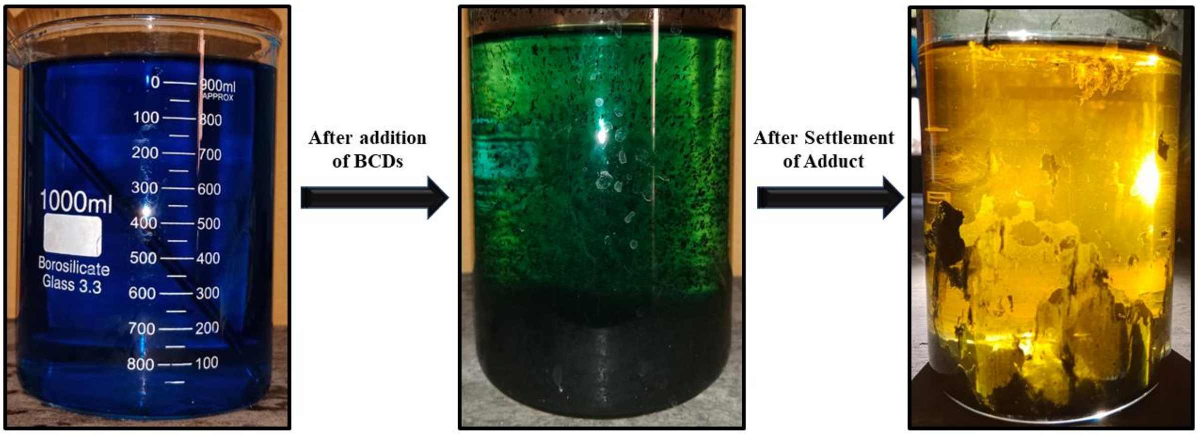

Fig. S5 Pictorial representation of one litre adsorption process of MB dye on BCDs

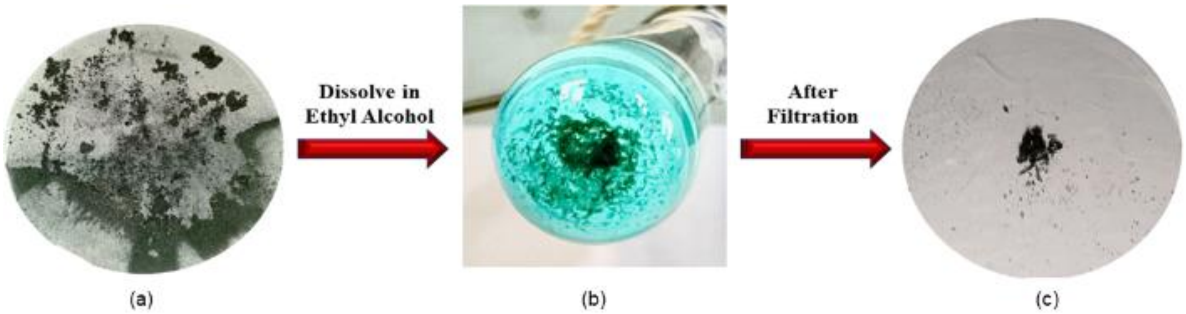

Fig. S6 Regeneration process of MB dye from the adduct (A) adduct (B) adduct after dissolving in ethyl alcohol (C) regenerated BCDs

Supplementary table ST1 effect of adsorbent dose

Concentration of adsorbent (Dilution factor)	Percentage of adsorption (%)
Stock Solution	20.09
1	23.60
2	44.53
4	47.44
6	53.09
8	57.67
10	64.70

Supplementary table ST2 effect of concentration of MB dye

MB concentration (mg L^{-1})	Adsorption capacity (Q_t) (mg g^{-1})	Percentage of adsorption (%)
1	81	44.66
2	140	70.25
6	396	49.59
10	605	64.70

Supplementary table ST3 Effect of pH Solution

Initial pH of dye	Adsorption capacity (Q_t) (mg g^{-1})	Percentage of adsorption (%)
3	450	56.80
4	401	48.61
5	520	62.65
6	440	58.00
7	605	64.70
8	334	40.18
9	424	50.04
10	419	49.45
11	416	51.73
12	456	55.22

Supplementary table ST4 Effect of Salts

**Name of the salt (10^{-3} M)**	**Adsorption capacity (Q_t) ($mg\ g^{-1}$)**	**Percentage of adsorption (%)**
NaCl	611	65.39
KCl	616	65.85
$PbNO_3$	614	65.69
$NiCl_2$	773	82.65
$CuSO_4$	746	79.75
$FeSO_4$	689	73.72
$HgSO_4$	671	71.73
CH_3COONa	683	73.10
$CdCO_3$	636	68.06
$BaCl_2$	794	84.95

Supplementary table ST5 effect of adsorption in real sample

**Name of the Salt**	**Adsorption capacity (Q_t) (mg g^{-1})**	**Percentage of adsorption (%)**
Tap Water	535	43.21

Supplementary table ST6 effect of stirring

**Time (min)**	**Adsorption capacity (Q_t) (mg g^{-1})**	**Percentage of adsorption (%)**
10	395	33.78
20	401	34.33
30	436	37.36
40	445	38.05
50	444	37.99
60	462	39.52
Infinite Time	599	51.19

Supplementary table ST7 effect of contact time

	Adsorption capacity (Q_t) (mg g^{-1})				Percentage of adsorption (%)			
**Time (min)**	**10**	**6**	**2**	**1**	**10**	**6**	**2**	**1**
1	252.14	246.42	90.71	55.71	45.51	31.60	30.83	26.35
2	282.85	245.71	95.71	60	48.02	35.45	33.20	26.27
3	283.57	305	97.14	60.71	48.74	35.54	33.59	32.62
4	301.42	256.42	97.14	59.28	48.74	37.77	32.80	27.42
5	304.71	257.85	100	60.71	50.17	42.70	33.59	27.57
6	355	282.85	97.85	60.42	49.10	44.49	33.99	30.25
7	347.85	286.42	99.28	60.42	49.82	43.59	33.99	30.63
8	355	294.28	99.28	59.28	49.82	44.49	32.80	31.47
9	360.71	308.57	98.57	59.28	49.46	45.21	32.80	33.00
10	358.57	308.57	99.28	59.28	49.82	44.94	32.80	33.00

Supplementary table ST8 effect of slow addition

Volume of BCDs (ml)	Time (min)	Percentage of absorbance (%)
0.5	5	16.62
1.0	10	41.47
1.5	15	52.90
2.0	20	55.50
2.5	25	56.34
3.0	30	55.01
3.5	35	53.44
4.0	40	48.66
4.5	45	50.66
5.0	50	49.03
5.0	Infinite time	60.75

Supplementary table ST9 effect of temperature and thermodynamical parameter

Temperature	Adsorption capacity (Q_t) (mg g^{-1})	Percentage of adsorption (%)	ΔG (J mol^{-1})
305	605	64.7	-10335.1
315	97.85	23.06	-5931.6
325	85.71	22.72	-5746.98
335	75	19.81	-5578.38
